# Cascade Dams and Seasonality Jointly Structure Gut Microbiome Biogeography in *Saurogobio punctatus*

**DOI:** 10.3390/microorganisms14040745

**Published:** 2026-03-26

**Authors:** Rongchao He, Kangtian Zhou, Jiangnan Ni, Zhenxin Chen, Chenyu Yao, Mei Fu, Hongjian Lü, Weizhi Yao

**Affiliations:** Aquatic Biodiversity Conservation Research Center, College of Fisheries, Southwest University, Chongqing 400716, China; rongchaohe15@gmail.com (R.H.); ktz0524@foxmail.com (K.Z.); nijiangnan0808@163.com (J.N.); lcinxin@163.com (Z.C.); ycy11262026@163.com (C.Y.); fumei@swu.edu.cn (M.F.); hongjianlv@swu.edu.cn (H.L.)

**Keywords:** cascade dams, seasonal hydrology, *Saurogobio punctatus*, gut microbiota, community assembly

## Abstract

Cascade dams fragment river habitats, but how seasonal hydrology modulates the biogeography and assembly of fish gut microbiota remains unclear. We surveyed gut bacterial communities of the omnivorous fish *Saurogobio punctatus* across 10 reaches separated by cascade dams in the Qijiang River during the wet (summer) and dry (winter) seasons using 16S rRNA gene amplicon sequencing. Sampling was synchronized among reaches to minimize temporal variability. Winter exhibited stronger differentiation among reaches and a steeper distance–decay pattern, and reach-scale environmental heterogeneity (especially dissolved inorganic nitrogen) was more stable under weak hydrodynamics. Null model analyses showed that stochastic processes dominated in summer, with dispersal-related processes and drift being prominent under high connectivity, whereas deterministic assembly increased in winter and was mainly associated with homogeneous selection. Compositionality-aware differential abundance analysis (ANCOM-BC2) identified 409 genera with a significant seasonal differential abundance after adjusting for reach (FDR *q* < 0.05). Random forest classification, used as a complementary prediction-oriented feature-ranking analysis, indicated higher reach discriminability in winter, with Nitrospirota ranking among the top features. PLS-PM indicated that α-diversity had the strongest direct association with β-diversity in the specified model, whereas spatial and environmental effects were linked to β-diversity mainly through indirect, α-diversity-mediated pathways. Biologically, α-diversity may reflect an integrative summary of the within-gut taxon pool shaped by host filtering and environmentally derived inputs (e.g., diet- and habitat-associated sources), which can influence the magnitude of between-reach compositional turnover. Together, these results show that seasonal hydrological regimes tune spatial turnover and assembly of fish gut microbiota in cascade-regulated rivers.

## 1. Introduction

Rivers serve as a critical conduit linking terrestrial and marine ecosystems and represent major global hotspots of biodiversity; their longitudinal connectivity sustains key ecological processes, including material transport, energy flow, and biotic migration [1,2,3]. However, the acceleration of global hydropower development has contributed to the widespread interception and fragmentation of rivers by artificial dams, severely compromising longitudinal connectivity and creating typically fragmented habitats [4,5]. Specifically, cascade dams and their operation not only physically obstruct migration corridors and gene flow among aquatic organisms, which precipitates the geographic isolation of populations but also fundamentally reshapes reach-scale hydrodynamics and nutrient transport regimes. These alterations to original hydrological conditions and ecological processes impose novel environmental stressors on aquatic biota within river ecosystems [6].

Against this backdrop, how fish gut microbial communities respond to cascade-induced habitat fragmentation has received increasing attention [7,8]. Gut microbial communities not only contribute to host nutrient metabolism, nutrient assimilation, and growth and development but also play crucial roles in maintaining immune homeostasis and enhancing resistance to environmental stressors [9,10,11]. Previous studies have shown that fish gut microbiota are jointly shaped by host filtering (e.g., host physiology and diet) and habitat-associated environmental sources (e.g., ambient microbial pools and local food resources), which together influence gut community composition [12,13,14]. In natural rivers, seasonal turnover is a dominant external driver of gut microbiota succession: temperature shifts regulate ectotherm metabolism and the gut microenvironment, while seasonal changes in primary productivity and prey (e.g., algae and benthic invertebrates) can alter diets of omnivorous fishes and restructure the gut microbiome [15,16]. However, in cascade-regulated rivers, dam operations superimposed on seasonal hydrology generate complex spatiotemporal heterogeneity, and how these interacting gradients shape fish gut microbiota remains insufficiently understood [17,18].

Habitat fragmentation induced by cascade dams and seasonal environmental fluctuations may jointly reshape the assembly trajectories of fish gut microbiota by altering the relative strengths of deterministic selection and stochastic dispersal [19]. On the one hand, reaches separated by dams often differ in hydrodynamic and material regimes (e.g., reduced flow and longer retention, altered nutrient availability and particle loads, and shifts in local food resources); these environmental gradients can impose distinct selective pressures on gut-associated microbes and contribute to reach-scale biogeographic differentiation, particularly when environmental filtering is strong [20,21,22]. On the other hand, the physical barrier imposed by dams truncates longitudinal connectivity and strengthens dispersal limitation of hosts and their associated microbiota, potentially increasing the relative contribution of stochastic processes such as ecological drift during community assembly and thereby amplifying inter-community β-diversity differences [23]. Nevertheless, quantitative field studies that integrate continuous sampling with ecological modeling to evaluate how cascade fragmentation and seasonal fluctuations jointly regulate these deterministic and stochastic processes in indigenous fish remain scarce [24,25,26,27].

The Qijiang River is an important tributary in the upper Yangtze Basin, where multiple cascade hydropower stations have been constructed along the mainstem, creating a characteristic river–reservoir alternating habitat and providing an ideal natural laboratory for disentangling the ecological effects of river fragmentation. *Saurogobio punctatus* is a native cyprinid fish in the Qijiang River Basin. It is a riverine species that typically occupies the mid to lower water column and frequently forages near the river bed. It is omnivorous, with animal prey constituting an important diet component [28], making it an excellent indicator organism for probing microecological response patterns in cascade-dammed rivers. In this study, we targeted *S. punctatus* in the Qijiang River Basin and established ten consecutive sampling reaches along the cascade-dammed continuum during the wet (summer) and dry (winter) seasons. This study integrated high-throughput 16S rRNA gene sequencing, distance–decay relationship (DDR) modeling, null model-based community assembly analyses, and machine learning algorithms (e.g., Random Forest) to characterize the spatiotemporal biogeographical patterns of the gut microbiota and quantify the relative contributions of ecological processes, such as environmental selection, dispersal limitation, and ecological drift. This study provides field evidence for the spatiotemporal responses and potential assembly drivers of gut microbiota in native fishes under cascade fragmentation and seasonal hydrological disturbance, with implications for aquatic ecological restoration and fisheries management in the Yangtze River Basin.

## 2. Materials and Methods

### 2.1. Study Area

**Figure 1:**
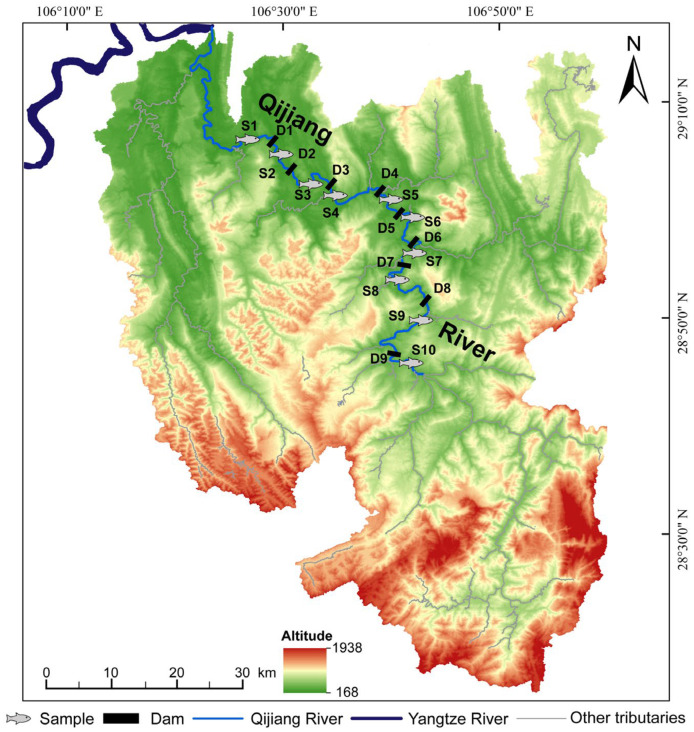
Map of the study area and sampling design in the cascade-dammed Qijiang River basin (Chongqing, China). The Qijiang River, a major right-bank tributary of the upper Yangtze River, is shown with nine cascade dams labeled D1–D9 (Table S1 for dam names, coordinates, and longitudinal distances) and ten consecutive sampling reaches (S1–S10) arranged from downstream (S1) to upstream (S10) along the mainstem to represent the longitudinal fragmentation gradient. The background shows basin elevation (altitude), with the Yangtze River and other tributaries indicated for geographic context.

The Qijiang River is a major tributary on the right bank of the upper Yangtze River and joins the Yangtze mainstem in Jiangjin District, Chongqing, China. Due to the construction of multiple cascade hydropower stations along the mainstem, the basin exhibits a characteristic river–reservoir alternating habitat. This study focused on the Chongqing reach of the Qijiang River, where cascade hydropower development is pronounced; this reach is 157.5 km long and drains a catchment area of 4751 km^2^. Ten consecutive sampling reaches were established along the cascade-dammed reaches from downstream to upstream (S1–S10, with S1 at the lowermost site and S10 at the uppermost) to represent the longitudinal gradient of habitat fragmentation (Figure 1). Dam names, geographic coordinates, and longitudinal distances for the cascade system (D1–D9) are provided in Table S1.

### 2.2. Sample Collection

This study was conducted under a special fishing permit issued by the local agricultural authority (No. (Yu) Changyukejian [2022] 41, China). Field surveys were carried out in June 2023 (summer, wet season) and December 2023 (winter, dry season), each lasting 5 days. Fish were collected at each reach using a standardized combination of fishing gears [29]. At each sampling site, the deployed gear included one three-layer floating gillnet (mesh 3 cm; 50 m × 3 m), three three-layer bottom-set gillnets (mesh 3, 6.3, 11.2 cm; each 50 m × 3 m), and two cage traps (8.0 m × 0.23 m × 0.32 m). Each sampling operation lasted 14 h (17:00–07:00 the next day).

Adult *S. punctatus* individuals with no visible external injuries and vigorous health were selected as target specimens; at each site, four adult individuals of comparable body size were randomly selected (*n* = 4) as biological replicates. The entire intestinal tract was aseptically dissected. The outer surface of the intestine was rinsed with sterile PBS to reduce exogenous surface contamination; whole-gut samples (including intestinal contents) were used to represent the overall gut microbial community. Processed intestinal samples were placed in 2 mL sterile cryovials, rapidly frozen in liquid nitrogen, and subsequently transferred to a −80 °C freezer for storage until sequencing analysis [30].

At each site, water temperature (Tem), dissolved oxygen (DO), pH, and electrical conductivity (EC) were measured in situ using a multiparameter water quality meter (YSI ProPlus, Yellow Springs, OH, USA). Chlorophyll-a (Chla) was determined using a handheld chlorophyll meter (Chloro Tech 121A, Boston, MA, USA). Water samples were transported to the laboratory under cold conditions, and total nitrogen (TN), total phosphorus (TP), ammonium nitrogen (NH_4_^+^-N), nitrate nitrogen (NO_3_^−^-N), nitrite nitrogen (NO_2_^−^-N), and the permanganate index (COD) were determined following standard methods [31]. Dissolved inorganic nitrogen (DIN) was calculated as DIN = NH_4_^+^-N + NO_3_^−^-N + NO_2_^−^-N. Environmental measurements were based on composite water samples per site (three subsamples pooled), and all physicochemical determinations were conducted in triplicate; mean values were used for downstream analyses (Table S2).

### 2.3. DNA Extraction and Sequencing

Total genomic DNA was extracted from whole intestinal samples using the E.Z.N.A.^®^ Soil DNA Kit (Omega Bio-tek, Norcross, GA, USA). DNA quality was assessed by 1% agarose gel electrophoresis and a NanoDrop 2000 spectrophotometer (Thermo Fisher Scientific, Waltham, MA, USA). The V3–V4 region of the bacterial 16S rRNA gene was amplified using the universal primer pair 341F (5′-CCTACGGGNGGCWGCAG-3′) and 806R (5′-GGACTACHVGGGTWTCTAAT-3′) [32]. Amplicons were purified and quantified, pooled at equimolar concentrations, and used for library preparation, followed by paired-end sequencing on an Illumina MiSeq platform (PE300) [33].

### 2.4. Bioinformatics and Statistical Analysis

Raw sequencing reads were first demultiplexed and quality-filtered; barcodes and primer sequences were removed, and low-quality reads were discarded. Paired-end reads were merged and then processed using the UPARSE pipeline for chimera removal and 97% similarity OTU clustering; high-quality sequences were clustered into operational taxonomic units (OTUs) at 97% sequence similarity, and a representative sequence was selected for each OTU. Taxonomic assignment of representative sequences was performed using the RDP Classifier against the SILVA reference database [34,35]. Software versions and key parameters. Raw reads were quality-filtered using fastp (v0.18.0) with the following criteria: (i) reads containing ambiguous bases (N) ≥ 10% were removed; (ii) reads in which bases with Phred quality ≤20 accounted for ≥50% were removed; and (iii) reads containing adapter sequences were discarded. Paired-end reads were merged into tags using FLASH (v1.2.11) with a minimum overlap length of 10 bp and a maximum mismatch rate of 2%. Low-quality tags were further filtered following a QIIME-style tag QC procedure: tags were truncated at the first base position where consecutive low-quality bases (default threshold Q ≤ 3) reached a specified run length (default = 3), and tags with continuous high-quality bases shorter than 75% of the tag length were removed. OTU clustering was performed using USEARCH (v9.2.64) with the UPARSE algorithm at 97% sequence similarity; chimeras were detected and removed using the UCHIME algorithm. The most abundant tag sequence within each OTU was selected as the representative sequence. Taxonomic assignment was conducted using the RDP classifier (v2.14) against the SILVA database (v138.1) with a confidence threshold of 0.8. To minimize non-target interference, sequences annotated as chloroplasts or mitochondria, non-bacterial lineages, and singleton OTUs were removed [36]. After quality filtering and chimera removal, per-sample sequencing depth (Effective Tags) ranged from 29,894 to 131,394 (median = 87,103). After this removal step, the final OTU table totals ranged from 18,394 to 113,414 counts per sample (median = 54,878) (Supplementary Data S4). To standardize sampling effort for alpha-diversity estimation and to assess sequencing coverage, OTU richness rarefaction curves were generated (Figure S1), and alpha-diversity metrics were calculated on an OTU table rarefied to 15,000 reads per sample by random subsampling without replacement; all samples exceeded this depth and were retained (*n* = 80).

Sequencing output and sample-level QC summary. Across all samples, 7,864,171 raw reads were generated. After quality filtering, 7,842,814 clean reads remained. After paired-end merging, 7,588,308 clean tags were obtained (per sample: mean = 94,854; median = 91,998; range = 30,704–134,161). After chimera removal, 7,317,155 effective tags remained (per sample: mean = 91,464; median = 87,103; range = 29,894–131,394). OTU clustering at 97% similarity yielded 11,453 OTUs. After removing non-bacterial lineages (including Archaea), chloroplast/mitochondria sequences, and singleton OTUs, 10,129 OTUs were retained for downstream community analyses. Detailed per-sample QC metrics and sequencing depth statistics are provided in Supplementary Data S4, and a summary of QC and OTU statistics is provided in Supplementary Data S5.

Normalization and data transformations. Alpha diversity was estimated on rarefied data (15,000 reads per sample; Figure S1). Relative abundance tables were generated from the OTU count table by total-sum scaling (TSS; counts divided by the per-sample total) and taxonomic aggregation to the target rank (e.g., phylum). For beta diversity, Bray–Curtis dissimilarities were calculated on TSS relative abundances, followed by PCoA, PERMANOVA, and betadisper. For constrained ordination (RDA) and envfit, TSS relative abundances were Hellinger-transformed prior to analysis to reduce the influence of highly abundant taxa. Random Forest classifiers were trained on phylum-level relative abundances derived as above (TSS proportions) and were used as a complementary, prediction-oriented analysis. Beyond the removal of non-bacterial lineages and chloroplast/mitochondria sequences and singleton OTUs described above, no additional low-prevalence/low-abundance filtering was applied for community-level analyses, except where explicitly stated (e.g., genus-level DA using ANCOM-BC2 with a prevalence filter). Input matrices were used in each module. Unless otherwise stated, abundance-based community analyses were conducted on the OTU-level community table after downstream filtering (removal of non-bacterial lineages, chloroplast/mitochondria sequences, and singleton OTUs) and without rarefaction. Specifically, Bray–Curtis dissimilarities were computed from OTU-level TSS relative abundances and used consistently for PCoA, PERMANOVA, betadisper, distance–decay (DDR), Mantel tests, and MRM. RDA/envfit was performed on the OTU-level community table after Hellinger transformation of TSS relative abundances. Random Forest classification used phylum-level features obtained by aggregating the OTU table to phylum and applying TSS (i.e., phylum-level relative abundances). Rarefaction (15,000 reads per sample) was applied only to alpha-diversity estimation and rarefaction-curve visualization.

All statistical analyses were conducted in R (v4.3.1), and data visualizations were primarily generated using the ggplot2 package [37]. Alpha-diversity indices (Shannon, Chao1, and Faith’s PD) were calculated using the vegan and picante packages. A phylogenetic tree required for Faith’s PD and phylogeny-based null-model analyses (β_NTI_) was constructed from OTU representative sequences and contains branch lengths with node support values ranging from 0 to 1. Prior to Faith’s PD and β_NTI_ calculations, the tree was pruned to match the OTUs retained in the final community table (10,129 OTUs) using R (e.g., ape/picante), and the pruned tree was then used to compute Faith’s PD and phylogenetic distance matrices for β_NTI_. A two-way ANOVA (Season, reach, and their interaction) was used to evaluate season × space interaction effects, and effect sizes were quantified as partial η^2^ using the effectsize package; longitudinal trends were tested using Spearman’s rank correlation [38]. Beta diversity was assessed using Bray–Curtis dissimilarity calculated on TSS relative abundances and visualized by principal coordinates analysis (PCoA). Differences in community structure and within-group dispersion were tested using PERMANOVA and betadisper, respectively [39]. Distance–decay relationships (DDRs) were evaluated using the number of dams between sites as a measure of spatial isolation, based on the pairwise Bray–Curtis community dissimilarity matrix [40]. Mantel tests were used to assess correlations between the Bray–Curtis community dissimilarity matrix and (i) the spatial-distance matrix defined by dam counts and (ii) environmental-distance matrices. For each environmental factor, values were z-score standardized within each season, Euclidean distance matrices were then computed, and Mantel correlations were performed against the Bray–Curtis matrix [41,42]. Multiple regression on distance matrices (MRMs) were used to jointly include spatial distance and multivariate environmental distance to evaluate their independent contributions, with permutation tests used to assess the significance of regression terms [43]. Community assembly processes were inferred using the null model framework based on β_NTI_ and abundance-weighted RC_Bray_. Specifically, β_NTI_ was used to quantify deviations in phylogenetic turnover, and RC_Bray_ was combined with β_NTI_ to partition stochastic versus deterministic processes [44,45]. In addition, the Sloan neutral community model (NCM) was applied to evaluate the contribution of stochastic processes [46]. Key environmental drivers were identified using redundancy analysis (RDA) on OTU-level community tables that were Hellinger-transformed from TSS relative abundances (season-stratified), and permutation tests were used to evaluate the significance of constrained axes and fitted environmental vectors [47]. Multiple-testing correction. Given the large number of parallel hypothesis tests across distinct inferential modules, we defined major test families and controlled the false discovery rate (FDR) using the Benjamini–Hochberg procedure within each family (*q* < 0.05 unless otherwise stated). Specifically, FDR correction was applied to (i) Mantel tests across multiple environmental variables within each season; (ii) envfit tests for multiple fitted environmental vectors within each season in the RDA framework; and (iii) bootstrap-based significance tests of PLS-PM structural paths within each seasonal model. For transparency, both nominal *p* values and FDR-adjusted *q* values are reported in the corresponding tables (Mantel: Table S6; envfit: Table S5; PLS-PM: Table 1). Differential abundance analysis (ANCOM-BC2) used BH-FDR adjustment as reported (Table S8; Supplementary Data S1–S3). To provide a formal, taxon-wise differential abundance (DA) assessment while accounting for compositionality and multiple testing, we performed genus-level DA analysis using ANCOM-BC2 on the non-rarefied OTU count table aggregated to the genus level. Rarefaction was applied only for diversity analyses and not for DA. Genera with low prevalence (present in <5% of samples) were filtered to reduce sparsity. For seasonal effects, we fitted a multivariable model including Season and Reach (Genus ~ Season + Reach). For reach-associated taxa, DA was evaluated within each season separately (Genus ~ Reach). ANCOM-BC2 reports bias-corrected log fold changes (LFCs) with Wald tests; *p* values were adjusted using the Benjamini–Hochberg false discovery rate (FDR) procedure (*q* < 0.05). DA results are summarized in Table S8, and the full ANCOM-BC2 output tables are provided as Supplementary Data S1–S3 [48,49]. As a complementary, prediction-oriented analysis, reach discriminability was evaluated using random forest classification implemented with the ranger engine via caret. Models were fitted separately for each season (*n* = 40 samples per season; 10 reaches with *n* = 4 replicates per reach), and class sizes were therefore balanced by design. Prior to modeling, near-zero-variance features were removed (caret::nearZeroVar). Model performance was assessed using a single 5-fold cross-validation, and overall accuracy was computed from out-of-fold predictions pooled across folds, accompanied by confusion matrices. Forests were trained with 1000 trees (num.trees = 1000) and mtry was tuned over a small candidate set (⌊sqrt(*p*)⌋, ⌊*p*/3⌋, ⌊*p*/2⌋) using splitrule = “gini” (min.node.size = 1). Variable importance was quantified using permutation importance (ranger importance = “permutation”). Because this is a 10-class problem (chance-level accuracy = 10%) with limited sample size, random forest results were interpreted as exploratory indicators of reach discriminability rather than as definitive biological evidence for reach-specific taxa [50]. This machine-learning analysis was used for classification and exploratory feature ranking, and was not treated as a substitute for formal DA testing. The SPEC–OCCU framework was also applied, and taxa with Spec ≥ 0.7 and Occ ≥ 0.7 were defined as specialists [51]. Partial least squares path modeling (PLS-PM) was employed to evaluate the direct and indirect effects of reach-based spatial structure (SPA) and environmental factors (ENV) on α- and β-diversity, with bootstrap resampling used to test the robustness and significance of path coefficients [52]. PLS-PM was fitted separately for each season using standardized variables. The α-diversity construct was represented by Shannon, Chao1, and Faith’s PD, and β-diversity was summarized using Bray–Curtis PCoA scores; SPA represents longitudinal position along the cascade (reach order), and ENV represents the measured physicochemical conditions at each reach.

## 3. Results

### 3.1. Spatiotemporal Patterns of Gut Microbial Diversity and Community Structure

**Figure 2:**
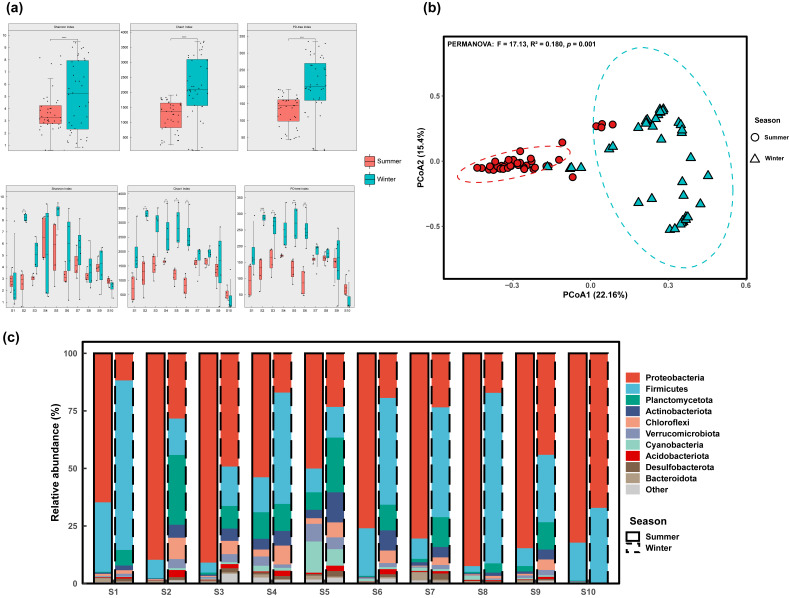
Spatiotemporal patterns of gut bacterial diversity, community structure, and phylum-level composition in *S. punctatus* across 10 cascade-dam reaches (S1–S10) of the Qijiang River. (**a**) α-diversity (Shannon, Chao1, Faith’s PD) in summer and winter. Significance is denoted as *p* < 0.05 (*), *p* < 0.01 (**), *p* < 0.001 (***), and *p* < 0.0001 (****). (**b**) Bray–Curtis PCoA (PERMANOVA: F = 17.13, R^2^ = 0.180, *p* = 0.001; 999 permutations). (**c**) Phylum-level relative abundance in summer and winter, computed from the OTU table via total-sum scaling (TSS) and phylum aggregation.

Two-way ANOVA showed significant interaction effects between season and reach on the alpha diversity of *S. punctatus* gut microbiota (*p* < 0.05, Table S3). In addition, spatial grouping explained a higher proportion of the variation in Shannon and Faith’s PD (Partial η^2^: 0.37–0.50), which was generally stronger than the main seasonal effects; Chao1 was influenced by both spatial and seasonal factors. Overall, the Chao1 and PD indices of the gut microbiota were significantly higher in winter (dry season) than in summer (wet season) (*p* < 0.001). In terms of spatial patterns, diversity high points shifted seasonally: in summer, high values were primarily found in the midstream (S4), while in winter, they were concentrated in the downstream (S2, S5). In addition, Chao1 and Faith’s PD decreased significantly from downstream to upstream in winter (Spearman ρ ≈ −0.49 to −0.50, *p* < 0.001), exhibiting a longitudinal gradient of “higher values downstream, lower values upstream”. The S10 reach exhibited the lowest Chao1 and Faith’s PD values in both seasons (Figure 2a; Table S4). These alpha-diversity metrics were calculated on the OTU table rarefied to 15,000 reads per sample (Figure S1).

PCoA based on Bray–Curtis dissimilarity revealed that samples were differentiated in community structure across both seasonal and spatial scales (Figure 2b and Figure S2). PERMANOVA confirmed that the seasonal effect was significant (F = 17.13, R^2^ = 0.180, *p* = 0.001; 999 permutations). Season-stratified PERMANOVA further indicated that reaches explained a substantial proportion of community-structure variation in both seasons, with a higher explanatory power in winter (Winter: R^2^ = 0.464; Summer: R^2^ = 0.417; *p* = 0.001; 999 permutations). The betadisper test detected no significant differences in within-reach dispersion in either season (Summer: F (9, 30) = 0.6527, *p* = 0.747; Winter: F (9, 30) = 0.8005, *p* = 0.631), suggesting that the PERMANOVA reach effect was unlikely to be associated with heterogeneity in dispersion.

At the phylum level, relative abundance profiles suggested that communities in both seasons were dominated by a few major phyla, with seasonally shifted contributions (Figure 2c). Because phylum-level summaries are descriptive, formal taxon-wise differences were evaluated using a compositionality-aware DA framework at the genus level; 409 genera showed significant seasonal differential abundance after adjusting for reach effects (ANCOM-BC2, FDR *q* < 0.05; Table S8; Supplementary Data S1).

### 3.2. Distance-Decay Patterns and Community Assembly Mechanisms

**Figure 3:**
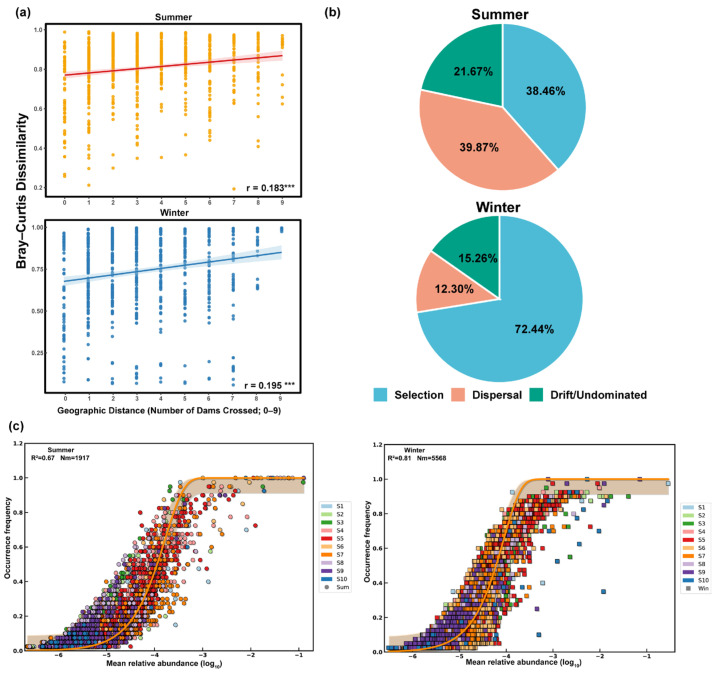
Distance–decay and assembly mechanisms of gut bacterial communities across the cascade-dam gradient. (**a**) Bray–Curtis dissimilarity increased with dam-distance (0–9 dams crossed) in both seasons (Mantel test, 999 permutations; Summer: r = 0.183, *p* = 0.001; Winter: r = 0.195, *p* = 0.002). (**b**) Seasonal shifts in assembly processes inferred from the β_NTI_–RC_bray_ null model (five processes summarized into selection, dispersal-related processes, and drift/undominated). (**c**) Neutral Community Model (NCM) fits of occurrence frequency versus mean relative abundance (log_10_), showing the best-fit curve with 95% confidence interval; R^2^ and Nm are reported for each season. Significance is denoted as *** *p* < 0.001.

The DDR analysis using the number of intervening dams (0–9) showed that Bray–Curtis dissimilarity of the *S. punctatus* gut microbiota increased with dam count, indicating a consistent distance–decay pattern and longitudinal community turnover along the cascade river system. This relationship was significant in both seasons (Summer: r = 0.183, *p* = 0.001; Winter: r = 0.195, *p* = 0.002; 999 permutations). The distance–decay slope was steeper in winter than in summer (Winter slope = 0.0193; Summer slope = 0.0110), whereas the overall explanatory power remained low (R^2^ = 0.034–0.038), suggesting that community assembly may also be influenced by additional unmeasured factors beyond physical isolation (Figure 3a).

Null model inference based on β_NTI_ combined with RC_Bray_ revealed a seasonal shift in community assembly processes (Figure 3b). In summer, stochastic processes predominated (61.5%), with dispersal limitation contributing 39.7% and drift (undominated processes) accounting for 21.7%; deterministic processes represented 38.5%, and were primarily associated with homogeneous selection (25.8%). In contrast, winter was dominated by deterministic processes (72.4%), with homogeneous selection being the most prevalent component (67.7%); meanwhile, dispersal limitation and drift accounted for 12.3% and 15.3%, respectively. Overall, the relative contribution of deterministic processes was higher in winter than in summer. Furthermore, the steeper distance–decay slope in winter suggested that, even when homogenizing selection-dominated phylogenetic turnover, compositional turnover along the cascade-dammed continuum remained detectable.

The Sloan NCM was used to characterize the relationship between OTU occurrence frequency and mean relative abundance (Figure 3c). Both seasons showed a good neutral fit, with a higher model fit in winter than in summer (Winter: R^2^ = 0.81; Summer: R^2^ = 0.67). The estimated effective migration parameter (Nm) was higher in winter (5568) than in summer (1917), indicating a higher estimated neutral-model immigration signal within the Sloan framework rather than a direct proxy for hydrological connectivity. This pattern was broadly consistent with the β_NTI_–RC_Bray_ inference showing a reduced contribution of dispersal limitation in winter, offering convergent (but not identical) support across frameworks that target different dimensions of assembly. In both seasons, a subset of OTUs fell outside the 95% confidence interval, suggesting that non-neutral processes (e.g., environmental filtering) also contribute to community assembly beyond stochastic dispersal.

### 3.3. Key Environmental Drivers of Gut Microbial Community Structure

**Figure 4:**
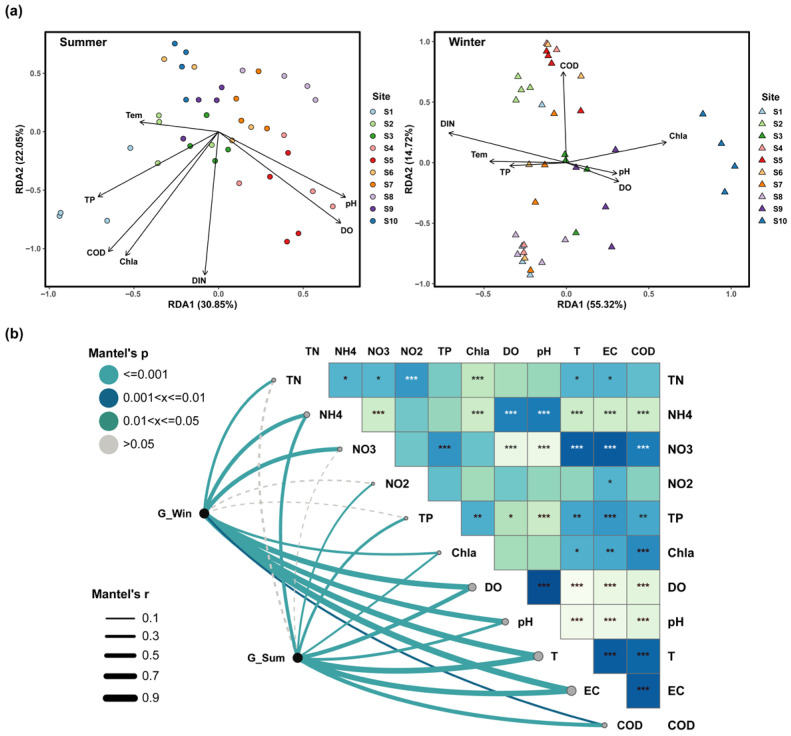
Environmental drivers of gut bacterial community structure. (**a**) Redundancy analysis (RDA) ordinations for summer (**left**) and winter (**right**) with fitted environmental vectors. (**b**) Mantel correlation network linking Bray–Curtis community dissimilarity to environmental variables in summer (G_Sum) and winter (G_Win); edge width is proportional to Mantel’s r and edge color indicates BH-FDR-adjusted *q* values within each season (legend). The heatmap shows pairwise correlations among environmental variables, with significance denoted by asterisks (* *p* < 0.05, ** *p* < 0.01, *** *p* < 0.001).

RDA constrained ordination combined with envfit (Monte Carlo permutation test, 999 permutations) showed that the associations between gut microbial community structure and environmental gradients differed between summer and winter. To account for multiple testing across fitted vectors, envfit *p* values were adjusted within each season using the Benjamini–Hochberg FDR procedure, and both nominal *p* values and FDR-adjusted *q* values are reported in Table S5. In summer, samples displayed a coherent directional separation in the RDA space along nutrient and organic load-related variables (COD, Chla, DIN, and TP) (Figure 4a, Table S5). Among these factors, COD, Chla, and DIN exhibited relatively strong and significant fits (r^2^ = 0.40–0.42) and remained significant after FDR correction (*q* < 0.05; Table S5). pH, DO, and TP also showed moderate fits (r^2^ = 0.26–0.36), and their significance should be interpreted based on the corresponding FDR-adjusted *q* values (Table S5). By contrast, temperature showed a weaker and non-significant association (Tem: r^2^ = 0.08) and was not supported after FDR correction. In winter, the environmental association signal was more focused: DIN showed the strongest fit (r^2^ = 0.54) and remained significant after FDR correction, followed by Chla (r^2^ = 0.37) (*q* < 0.05; Table S5). Tem and COD showed weaker fits (r^2^ = 0.18–0.21) and their significance should be interpreted based on the corresponding FDR-adjusted *q* values, whereas TP, DO, and pH were not supported after FDR correction. Overall, summer exhibited a multifactor pattern with jointly associated environmental drivers, whereas winter was primarily aligned with directional filtering along the DIN gradient, with secondary contributions from Chla, Tem, and COD.

Mantel test results further corroborated the patterns identified above. After controlling the false discovery rate across tested environmental variables within each season (BH-FDR; Table S6), the Bray–Curtis community dissimilarity matrix remained significantly correlated with Tem and EC in both seasons (Summer: r = 0.69 and 0.66; Winter: r = 0.71 and 0.76; *q* < 0.01), and correlations with DO and Chla also remained significant in both seasons (*q* < 0.01). Environmental correlates also showed seasonal differences after FDR correction: NO_2_^−^-N and TP were significant only in summer, whereas TN and NO_3_^−^-N were significant only in winter (Figure 4b; Table S6). Notably, the winter-specific association with NO_3_^−^-N was consistent with the RDA results in which DIN showed the strongest fit. It should be noted that RDA and Mantel tests identified partially overlapping sets of key variables, reflecting differences in their statistical foundations. Thus, although DIN exhibited a stronger linear fit in the RDA framework, the high Mantel correlations observed for other significant variables (e.g., Tem and EC) may indicate their roles as broader background gradients that constrain β-diversity distances in a more complex manner, rather than acting as single linear drivers.

### 3.4. Signature Taxa Driving Spatiotemporal Community Differentiation

We further conducted genus-level differential abundance (DA) analysis using ANCOM-BC2 to provide a statistically grounded assessment of taxon-level differences while accounting for compositionality. After FDR correction, 409 genera showed significant seasonal differential abundance in the multivariable model adjusting for reach effects (*q* < 0.05; Table S8; Supplementary Data S1), with more genera showing higher abundance in winter (232; LFC > 0) than in summer (177; LFC < 0). For example, *Aeromonas* (LFC = 3.83, *q* = 3.00 × 10^−14^) and *Mycoplasma* (LFC = 3.81, *q* = 3.67 × 10^−8^) were higher in winter, whereas *Pseudomonas* (LFC = −5.36, *q* = 2.76 × 10^−18^), *Enterobacter* (LFC = −4.93, *q* = 3.66 × 10^−20^), and *Escherichia–Shigella* (LFC = −4.85, *q* = 1.10 × 10^−18^) were higher in summer. Within-season reach-level DA further identified 112 (summer) and 24 (winter) genera showing significant reach-associated differences based on the global test (*q* < 0.05; Table S8; Supplementary Data S2 and S3).

**Figure 5:**
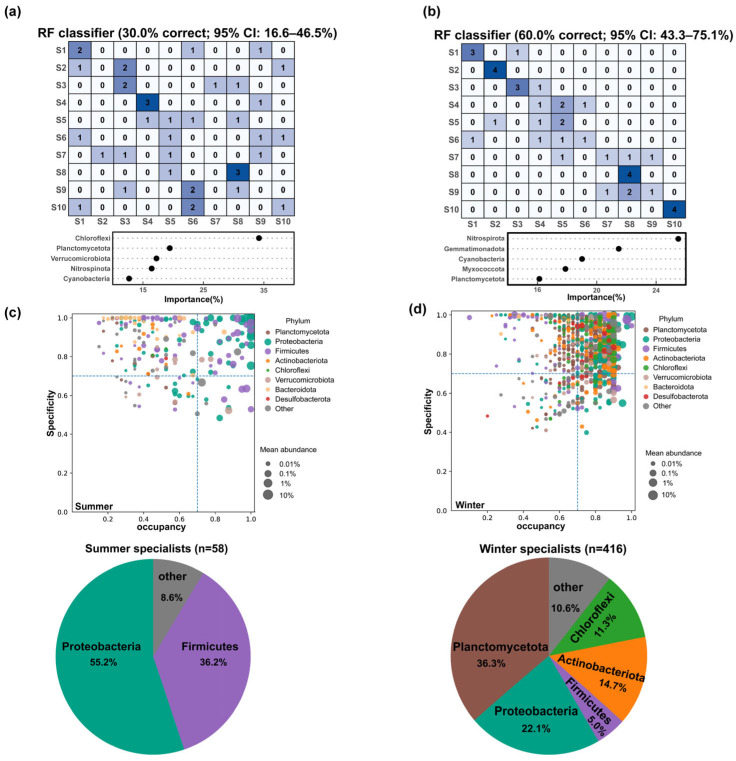
Seasonal differences in reach discriminability and SPEC–OCCU patterns of *S. punctatus* gut microbiota. (**a**,**b**) Random Forest classifiers predicting reach identity (S1–S10) from phylum-level relative abundances derived from the OTU table via total-sum scaling (TSS) and phylum aggregation in summer (**a**) and winter (**b**); confusion matrices show predictions, with darker blue shading indicating higher classification counts, and dot plots show the top phyla by variable importance. Model performance was evaluated by 5-fold cross-validation and variable importance was permutation-based. (**c**,**d**) OTU-level occupancy (Occ)–specificity (Spec) relationships in summer (**c**) and winter (**d**); point size denotes mean relative abundance and colors denote phyla. Dashed lines indicate the specialist thresholds (Occ ≥ 0.7, Spec ≥ 0.7), and pie charts summarize the phylum composition of seasonal specialists (Summer: *n* = 58; Winter: *n* = 416).

As a complementary, prediction-oriented analysis, we used Random Forest classification to quantify how well phylum-level profiles discriminate reach identity and to provide an exploratory feature importance ranking (Figure 5a,b); this analysis does not constitute formal DA testing, and the models do not provide formal evidence for reach-specific biomarker taxa. In summer, the phylum-level model achieved an overall accuracy of 30.0% based on 5-fold cross-validation (12/40 correct; 95% CI: 16.6–46.5%; Figure 5a), with extensive cross-misclassification; only S4 and S8 reached 3/4 correct, whereas four reaches (S2, S6, S7, and S9) had 0/4 correct, indicating weak reach separability at this taxonomic resolution. In winter, accuracy increased to 60.0% based on 5-fold cross-validation (24/40 correct; 95% CI: 43.3–75.1%; Figure 5b), substantially above the 10% chance-level baseline; reaches S2, S8, and S10 were predicted with high accuracy (4/4 correct each), and misclassifications occurred more frequently between neighboring reaches (50% of errors were to adjacent reaches), notably among S4–S6 and S8–S9. Nitrospirota ranked highest in winter feature importance based on permutation importance, followed by Gemmatimonadota, Cyanobacteria, Myxococcota, and Planctomycetota; this ranking reflects an association with reach discriminability in the fitted model and should be interpreted cautiously given the known instability of importance rankings in multi-class settings. To further interpret seasonal differences in reach discriminability suggested by Random Forest, we applied the SPEC–OCCU framework to quantify OTU-level specificity (Spec) and occupancy (Occ), and defined specialists as taxa with Spec ≥ 0.7 and Occ ≥ 0.7. At the seasonal scale, winter specialists (*n* = 416) outnumbered summer specialists (*n* = 58), and winter OTUs were more skewed toward higher-occupancy values (Occ ≥ 0.7), indicating a greater number of high-occupancy OTUs meeting the specialist thresholds in winter (Figure 5c,d). This seasonal asymmetry was directionally consistent with the null model results showing a higher contribution of deterministic processes in winter. At the seasonal scale, summer specialists were dominated by Proteobacteria (55.2%) and Firmicutes (36.2%), whereas winter specialists were dominated by Planctomycetota (36.3%) and Proteobacteria (22.1%), with additional contributions from Actinobacteriota (14.7%) and Chloroflexi (11.3%); Firmicutes accounted for 5.0%, and other phyla comprised 10.6%. When specialists were quantified at the reach level (i.e., specialists identified within each reach separately across the spatial gradient S1–S10), their spatial concentration patterns differed between seasons (Figure S3). In summer, 282 specialists were identified (2.46%), but they were highly concentrated in the midstream reaches S4 (161; 57.1%) and S5 (56; 19.9%), together accounting for approximately 77.0% of all summer specialists; this spatial concentration may be consistent with, and could partly contribute to, the relatively low reach-level separability observed for the summer Random Forest model, although this inference is exploratory. In winter, 200 specialists were identified (1.75%), and their contributions were more spatially dispersed and broadly distributed, primarily originating from S5 (27.5%), S2 (18.5%), and S6 and S10 (11.0% each), consistent with the along river continuity and neighbor-confusion patterns observed in the winter Random Forest results. At the reach level, taxonomically, summer reach-level specialists were mainly contributed by Planctomycetota (22.3%) and Firmicutes (18.4%), whereas winter reach-level specialists were dominated by Proteobacteria (24.5%), with additional contributions from Firmicutes, Planctomycetota, and Actinobacteriota. Overall, the season- and space-dependent patterns revealed by SPEC–OCCU at the OTU level were broadly aligned with the enhanced reach separability observed in winter at the phylum level using random forests.

### 3.5. Spatiotemporal Stability of the Core Microbial Community

**Figure 6:**
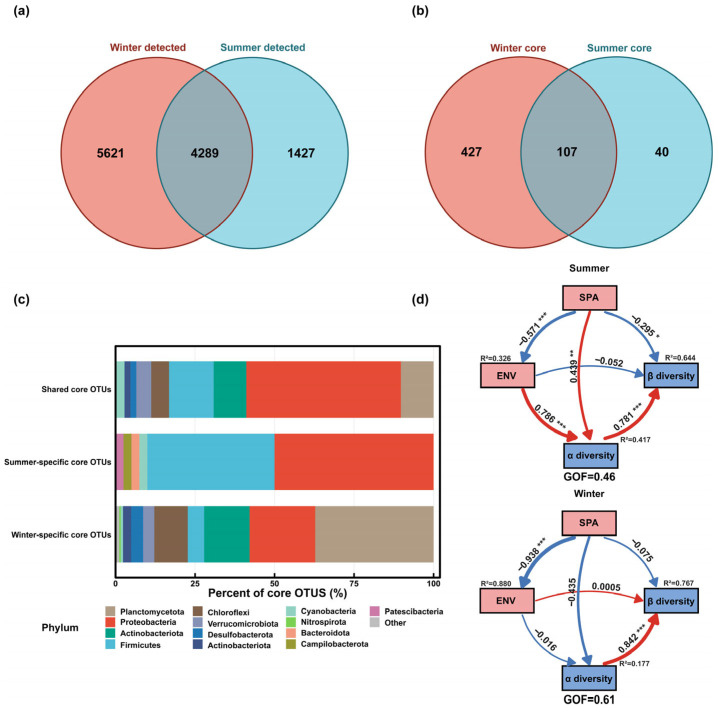
Seasonal turnover of detected and core gut OTUs, their taxonomic composition, and path-based quantification of drivers of community variation. (**a**) Venn diagram of detected OTUs in summer and winter. (**b**) Venn diagram of core OTUs (occurrence frequency, Occ ≥ 0.7) in each season. (**c**) Phylum-level composition of OTUs in the shared core, summer-specific core, and winter-specific core fractions. (**d**) Partial least squares path modeling (PLS-PM) constructed separately for summer and winter to evaluate direct links among reach-based spatial structure (SPA), environmental module (ENV), α diversity, and β diversity. Values on arrows are standardized path coefficients (red = positive, blue = negative); significance is indicated by asterisks based on BH-FDR-adjusted *q* values. R^2^ is reported for endogenous constructs (ENV, α diversity, β diversity), and GOF summarizes model fit. Significance is denoted as * *q* < 0.05, ** *q* < 0.01, and *** *q* < 0.001.

Analyses based on OTU detection and occupancy characteristics showed that both the detectable OTU pool and the core OTU pool were larger in winter than in summer. A total of 5716 OTUs were detected in summer and 9910 in winter, with 4289 OTUs shared between seasons; winter- and summer-specific OTUs numbered 5621 and 1427, respectively (Figure 6a). Using Occ ≥ 0.7 to define within-season core OTUs, the summer core set contained 147 OTUs, expanding to 534 in winter, with only 107 core OTUs shared between seasons (Figure 6b). Shared core OTUs accounted for 72.8% of the summer core set (107/147) but only 20.0% of the winter core set (107/534). Conversely, winter-specific core OTUs constituted 80.0% of the winter core set (427/534), exceeding the proportion of summer-specific core OTUs in the summer core set (37.4%, 40/147). These patterns indicate that, under the Occ ≥ 0.7 criterion, a larger fraction of OTUs maintained high occupancy within winter, implying higher within-winter repeatability; however, the limited cross-season overlap (20.0% of the winter core) also indicates substantial seasonal turnover of the core set.

At the phylum level, shared core OTUs were dominated by Proteobacteria (48.1%), with additional contributions from Firmicutes (13.9%), Actinobacteriota (10.2%), and Planctomycetota (10.2%) (Figure 6c). Summer-specific core OTUs were largely composed of Proteobacteria (50.0%) and Firmicutes (40.0%), whereas winter-specific core OTUs were mainly represented by Planctomycetota (37.2%), Proteobacteria (20.6%), Actinobacteriota (14.3%), and Chloroflexi (10.5%). Overall, the seasonal differences in core OTUs were consistent with the previously observed increase in deterministic-process contributions and the larger number of specialists in winter.

PLS-PM further disentangled the pathways through which reach-based spatial structure (SPA) and the environmental module (ENV) were associated with community variation (Figure 6d). In both seasons, α diversity showed a significant positive path coefficient to β diversity (Summer: 0.781, *q* < 0.001; Winter: 0.842, *q* < 0.001), and β diversity was well explained by the models (Summer R^2^ = 0.644; Winter R^2^ = 0.767). Model goodness-of-fit was higher in winter (GOF = 0.61) than in summer (GOF = 0.46). In the summer model, ENV had a significant positive effect on α diversity (coef = 0.786, *q* < 0.001), and SPA was negatively associated with ENV (coef = −0.571, *q* < 0.001). SPA also retained weak but significant direct paths to α diversity (coef = 0.439, *q* < 0.05) and β diversity (coef = −0.295, *q* < 0.05). By contrast, the winter model was more parsimonious: aside from a strong negative association between SPA and ENV (coef = −0.938, *q* < 0.001) and the positive link between α- and β diversity (*q* < 0.001), no other structural pathways were supported after BH-FDR correction (Table 1). This suggests that the explainable structure of winter community variation was primarily associated with the tight SPA–ENV linkage and the α diversity to β diversity relationship. Results from MRM are provided as robustness checks in the Supplementary Materials (Table S7), showing season-specific contributions of spatial and environmental distances to gut microbial β-diversity.

## 4. Discussion

Within the longitudinal river continuum reshaped by cascade dams, the gut microbiota of *S. punctatus* exhibited discernible spatial differentiation at the reach scale and showed a consistent response to seasonal hydrological context. This suggests that variation in gut communities across reaches and seasons cannot be explained by host-intrinsic processes alone and is instead consistent with joint influences from host filtering and environmentally mediated inputs. Under the interacting context of “fragmentation by cascade dams × seasonal hydrology,” a detectable and consistent along-river turnover of gut communities was observed across the surveyed reaches; however, spatial structuring was more pronounced in the dry season (winter), as reflected by higher explained variation and clearer longitudinal differentiation than in the wet season (summer). This seasonal contrast points to hydrodynamic connectivity as a potential regulator of the observed pattern: Higher summer discharge likely dilutes reach-scale physicochemical contrasts and synchronizes external source signals, thereby reducing the detectability of systematic between-reach differentiation, rather than implying unrestricted microbial mixing. This interpretation aligns with the view that fish gut microbiota are jointly shaped by environmental source pools and niche-based factors [53]. Overall, in this distinctive cascade-river system, our findings highlight the relative importance of seasonal shifts in connectivity in modulating fish gut community assembly, which may in turn alter the strength and detectability of fragmentation signals in the gut microbiome.

### 4.1. Seasonal Hydrology Reshapes the Spatial Structure and Assembly Mechanisms of S. punctatus Gut Microbiota

In the dry season (winter), the gut microbiota of *S. punctatus* showed clearer spatial structuring across reaches. Season-specific PERMANOVA indicated that reach explained more variation in winter than in summer, and this contrast was unlikely to be associated with differences in within-group dispersion. This pattern is consistent with a common expectation in river ecology that hydrodynamics regulate connectivity and mixing intensity, which in turn shapes the synchrony of external inputs. During the wet season, high discharge and strong hydrodynamic forcing can intensify advective transport and external inputs, which may dilute local physicochemical contrasts and make reach-scale environmental signals more similar, thereby weakening systematic between-reach differentiation [54]. By contrast, reduced water exchange during the dry season can allow environmental gradients to establish and persist across reaches. Against the background of host filtering, this increased external environmental heterogeneity may be more readily reflected in gut communities, promoting more repeatability along river differentiation trajectories [53]. Notably, winter also exhibited a clear downstream-to-upstream decline in α-diversity (Chao1 and Faith’s PD). A plausible explanation is that, under dry-season regulation, downstream reaches may experience more reservoir-like conditions and longer effective residence time, which may favor the retention and transformation of solutes and particles and promote the establishment of longitudinal resource gradients. Consistent with this interpretation, EC and NO_3_^−^-N were enriched in downstream reaches in winter, suggesting stronger solute accumulation and/or more complex external inputs along the cascade. Against the background of host filtering, these reach-scale differences may broaden habitat- and diet-associated source pools for gut colonization and be associated with a broader effective within-gut taxon pool downstream, whereas upstream reaches may experience stronger flushing and narrower resource spectra, resulting in lower within-gut diversity [54,55,56]. We treat these as mechanistic interpretations consistent with our field patterns and suggest that they should be further evaluated via direct diet/prey profiling and higher-resolution sequencing.

The DDR provides direct quantitative support for the interpretation above: although Bray–Curtis dissimilarity increased significantly with the number of intervening dams in both seasons, the DDR slope was steeper in winter. This suggests that under low flow conditions, the physical barrier effects of dams may amplify along river turnover signals, resulting in a steeper DDR slope. Notably, the overall explanatory power of the DDR models was low, suggesting that reach level-differences in the gut microbiota were not explained solely by geographic isolation but instead reflected the combined influence of water-quality gradients, food resources, and host physiology. This is consistent with findings from many disturbed rivers, where distance effects are detectable yet modest, and communities are jointly shaped by environmental conditions and external inputs [55,56].

Null model analyses further indicate a systematic seasonal shift in the assembly processes of the gut communities of *S. punctatus* under contrasting summer and winter hydrological contexts. Specifically, both the process attribution framework based on β_NTI_ and RC_Bray_ and the fit of the neutral community model point to the following seasonal differences: During the dry season, weaker hydrodynamic disturbance and a more stable environmental setting may impose more consistent and persistent selective constraints, increasing the predictability of community assembly and appearing as a higher contribution of homogenizing selection in the process attribution results. Against this backdrop, along river barriers and unmeasured differences in inputs can still superimpose a detectable signal of spatial turnover. This suggests that when physical disturbance weakens, local environmental factors (such as specific nutrient concentrations or food types) become a major force selecting for locally adapted microbial taxa. In the wet season (summer), intensified hydrodynamic disturbance and more synchronous external inputs likely increase the overall contribution of stochasticity. Notably, our process partitioning suggests that summer stochastic turnover is dominated by dispersal limitation and drift, indicating that reduced reach-scale distinguishability reflects a weaker systematic spatial signal rather than unrestricted (homogenizing) dispersal [24]. It is important to note that the goodness of fit of the NCM quantifies how closely the occurrence frequency to abundance relationship matches neutral expectations, and it does not necessarily negate the deterministic selection inferred from β_NTI_ and RC_Bray_. Rather, the two approaches can be viewed as complementary descriptions of community assembly. β_NTI_ and RC_bray_ emphasize deviations in turnover and the attribution of assembly processes, whereas the NCM focuses on whether the occurrence frequency to abundance relationship approaches neutral expectations. Because these approaches target different dimensions, they do not have to yield conclusions in the same direction. A similar seasonal switch, with stronger deterministic influence in the dry season and greater stochastic or dispersal related influence in the wet season, has been repeatedly reported for river bacterial communities [55,56]. Our results extend this pattern to the gut microbial system of *S. punctatus*, providing additional support. In summary, although fish gut microbiomes are continuously shaped by host filtering and niche processes within the gut, microbial communities in the surrounding water and sediment, together with their environmental conditions, may substantially alter the composition and structure of gut communities [57,58]. Therefore, seasonal fluctuations in hydrological connectivity and environmental gradients can manifest as differences in the spatial organization of the gut microbiome at the reach-scale, indicating that the gut microbiome of *S. punctatus* shows a detectable response, and some degree of plasticity, to the external hydrological and environmental context.

### 4.2. Seasonal Environmental Gradients Shape the Functional Implications of the Gut Microbiota and the Stability of Core Communities

The null model results indicate that gut community assembly in the dry season is dominated by deterministic processes, suggesting that key environmental gradients in this period may impose directional filtering on the gut microbial system. In our study, winter RDA and envfit identified DIN as one of the strongest explanatory factors in the constrained ordination. Mantel tests further suggest that community dissimilarity is correlated with multiple physicochemical variables; among them, nitrogen form indicators (DIN and NO_3_^−^-N) showed a consistent signal, aligning with the dominant DIN gradient observed in winter. These results suggest that the relatively stable hydrological retention conditions in the dry season may facilitate the establishment and persistence of along river chemical gradients, and are statistically consistent with stronger selective constraints. Although EC and Tem also showed correlations, considering both explanatory strength and ecological interpretability, DIN is better viewed as a plausible candidate axis (or correlate) capturing winter filtering gradients, rather than a single direct driver; EC and temperature may covary with nitrogen forms and jointly represent a broader constraint background in the dry season. At the same time, EC and temperature may covary with nitrogen form gradients, jointly forming a broader background constraint during the dry season. This view is consistent with recent river and reservoir studies showing that cascade reservoir operation can alter the transport and retention of biogenic elements and can reshape microbial taxonomic and functional composition at broader scales [6,59]. These studies also suggest that environmental filtering may be more pronounced in shaping biogeographic patterns during the dry season. Within this broader winter filtering context, the random forest results are best viewed as exploratory support for stronger reach discriminability, rather than as formal evidence for reach-specific biomarker taxa. Consistent with this pattern, Nitrospirota showed the highest permutation importance for reach classification in winter. Given that Nitrospirota lineages are often key nitrogen cycling taxa in sediments or biofilms, including roles in nitrite oxidation [60], and considering the foraging habits of *S. punctatus*, we speculate that the increased prominence of Nitrospirota in winter gut profiles (Figure 2c) and in the winter feature importance ranking (Figure 5b) may be compatible with external microbial inputs associated with feeding, rather than a direct in-gut response to dissolved nutrients. In this way, its statistical association can be consistent with the winter DIN gradient and with spatial structuring of near bed habitats. Therefore, in this study, Nitrospirota may serve as a candidate indicator associated with winter reach discriminability, although the underlying environment–food–host linkage remains hypothetical. This pattern is consistent with, but does not establish, an environment–food–host pathway: as a river dwelling omnivore, *S. punctatus* is unlikely to interact directly with dissolved nutrients in the water column through its gut microbiome, but may instead be linked to environmental conditions through diet and incidental ingestion of external microbes associated with near bed environments. Reservoir impoundment across cascade reaches may alter near bed conditions, including gradients in nitrogen forms, which could reshape the community composition of near-bed prey communities (e.g., benthic invertebrates) or their resource context and, under host filtering, lead to differentiated gut communities. Because taxonomic profiles based on the 16S rRNA gene do not directly equate to active functions, the mechanisms proposed above should be treated as working hypotheses and require further validation using metagenomics, functional genes such as *amo* or *nxr*, or metabolomic evidence [61,62].

More importantly, the directional filtering in winter was not confined to a few indicator taxa but concurrently reorganized the overall division of labor between the core community and specialist taxa. Core microbial communities are commonly viewed as the integrated outcome of host filtering and constraints imposed by the gut niche, representing stable members with high occupancy within the system [63,64]. In this study, the winter core community expanded markedly, reaching approximately 3.6 times the size observed in summer, and the number of specialists increased sharply (winter 416 vs. summer 58). This contrast suggests that the more stable environmental and resource context in the dry season may provide microbes with more persistent local niches, allowing more taxa to attain higher occupancy and enter a stable occurrence range, thereby increasing the predictability of community structure.

To disentangle the complex pathways, PLS-PM provides additional evidence at the level of path structure. Under the current PLS-PM specification, α-diversity emerged as the key proximal predictor of β-diversity; in this setup, ENV/SPA effects were captured mainly via diversity-related paths (more evident in summer), rather than through a single consistently significant direct path. This suggests that the effects of environmental and spatial factors on community differences are more likely expressed by shaping local diversity and the baseline level of community heterogeneity, rather than through a single direct pathway. Similar mediation frameworks have been used to disentangle direct and indirect effects in aquatic microbial systems [65,66]. In addition, the winter model showed better fit and a stronger linkage between SPA and ENV, indicating that environmental gradients in the dry season are more readily organized along spatial structure and can yield a clearer explanatory framework in statistical terms. This is consistent with reports that stronger signals of environmental filtering are more likely to emerge in winter [67]. Related reviews also emphasize that gut community structure is jointly shaped by the host and the surrounding habitat. Microbes from environmental sources can enter continuously through feeding and habitat contact and, under host filtering, form differentiated communities. In this context, the SPA–ENV linkage observed here can be interpreted as follows: during the dry season, spatially structured environmental and resource conditions may influence external microbial sources and nutrient availability, thereby indirectly strengthening the mediating role of α diversity for β diversity. Consequently, community differences may be more clearly organized along this statistical gradient, from spatially structured environmental context to diversity-related variation and then to dissimilarity [68,69]. From an ecological perspective, α-diversity may bridge reach-level habitat differences and community turnover because it reflects the effective within-gut taxon pool that can vary with diet resources and habitat-associated microbial sources (e.g., water, sediment, and biofilms) across fragmented reaches. Taken together, our results align with a framework in which environmental gradients are more stable in the dry season, filtering signals are clearer, and community predictability is enhanced. They also suggest that responses in the organization of core and specialist taxa, together with functional indicator groups, may jointly amplify biogeographic differentiation at the reach scale.

Beyond the Qijiang River, habitat- and geography-associated gut microbiome structuring has also been documented in other riverine fishes, providing context for the generality and boundary conditions of the “external inputs × host filtering” framework. For example, in wild redbelly tilapia (*Coptodon zillii*) sampled across contrasting habitats in a large subtropical river, habitat variation—including reaches influenced by cascade dams—was associated with marked gut microbiome differentiation and changes in α-diversity, highlighting the importance of reach-scale habitat context for gut microbiome assembly [70]. In addition, wild migratory black Amur bream (*Megalobrama terminalis*) showed pronounced gut community shifts across geographically isolated populations and seasons, with taxonomic changes linked to environmental gradients such as temperature and salinity [71]. Dam-altered river environments have also been discussed for endemic cyprinids; for instance, a study on *Rhinogobio cylindricus* in the Yangtze River suggested that host filtering may buffer or reshape environmental microbial signals under dam regulation [72]. Together, these studies align with our observation that reach-scale biogeography of the *S. punctatus* gut microbiome becomes more detectable under dry-season conditions, while the magnitude and direction of responses remain contingent on host ecology and the specific hydrological context of regulated rivers. From a management perspective, our results suggest that dam operations may modulate the detectability and strength of reach-scale habitat heterogeneity experienced by native fishes, particularly during the dry season. Cascade regulation could therefore be optimized to reduce extreme longitudinal discontinuities by maintaining minimum environmental-flow releases that support longitudinal exchange, avoiding prolonged low-flow stagnation within individual impoundments when feasible, and coordinating operational schedules among adjacent dams to minimize abrupt hydrological discontinuities along the mainstem. In addition, integrating fish gut microbiome indicators with routine physicochemical monitoring may provide a complementary, biologically integrated signal for diagnosing reach-specific habitat shifts under regulated flow regimes.

Although this study provides convergent support across multiple analytical frameworks, the scope of inference should be defined cautiously. First, this study is based on field observations and correlation-based models; therefore, the stronger winter covariation between gut communities and environmental gradients (including DIN) should be interpreted as pattern evidence that supports hypotheses, rather than direct causal proof. Unmeasured covariates, such as diet composition and availability, seasonal host physiological rhythms (e.g., reproductive activity, metabolic state, stress and immune status), and microbe–microbe interactions, may contribute to or confound the observed associations. Second, in cascade-regulated rivers, reach-based spatial structure (e.g., dam number) and local environmental differences are partly intertwined because dam operations can simultaneously reshape hydrodynamics and biogeochemical conditions. Accordingly, regression components (e.g., in MRM or PLS-PM) should be viewed as statistical contributions under the chosen variable sets, rather than fully separable “pure” space versus “pure” environment mechanisms. Third, the proposed “environment–food–host” cascade is a working hypothesis. We did not quantify gut contents or diet to verify seasonal or spatial shifts in food resources, and we did not concurrently profile water and sediment microbial communities to trace the transfer efficiency of key taxa from the environment to the gut. Future work combining diet or gut-content analysis (e.g., DNA metabarcoding or stable isotopes), environment–host source tracking (e.g., SourceTracker), and metagenomics or metabolomics will be needed to test this pathway explicitly. In addition, taxonomic profiles based on the 16S rRNA gene cannot be directly equated with active functional processes; therefore, inferences related to DIN mechanisms require validation using metagenomics, functional genes such as *amo* or *nxr*, and metabolomic or process rate evidence. Moreover, community profiling in this study was performed using 97% OTU clustering (97% sequence similarity) to maintain comparability with widely used OTU-based datasets and prior studies. We acknowledge that ASV-based denoising can provide finer taxonomic resolution; therefore, future work will reprocess the raw reads using an ASV pipeline to refine fine-scale patterns and evaluate whether higher-resolution profiling yields additional insights. Finally, because of constraints associated with field capture, biological replication within each reach-by-season combination was limited (*n* = 4), which may reduce the detection of low-abundance taxa and the statistical power for some effects and may limit evaluation of how well the machine-learning models generalize to future years or to reaches not included in sampling. Nonetheless, the main spatial and seasonal patterns were supported in a consistent direction across analytical approaches, suggesting that these limitations did not alter the central conclusions. This study focused only on *S. punctatus*; given differences among fish niches in habitat use and feeding strategies, the ways in which cascade dams shape gut microbiota may differ across species. Future studies should expand to multiple ecological niches and incorporate higher temporal resolution and repeated sampling across years.

## 5. Conclusions

Using the widely distributed omnivorous fish *S. punctatus* in the Qijiang River as a model, we show that reach-based spatial structures associated with cascade dams and seasonal hydrological rhythms can jointly shape the gut microbial system of a river-dwelling fish that typically occupy the mid-to-lower water and frequently forage near the river bed. Our results indicate that the response of gut microbiota to spatial barriers is not a fixed, uniform effect but is strongly modulated by seasonal environmental gradients. In the dry season (winter), weaker hydrodynamic conditions favor the stable emergence of between-reach environmental differences, exemplified by gradients in nitrogen forms, which strengthens deterministic filtering and leads to clearer spatial structuring and a steeper distance decay pattern. Mechanistically, dry-season environmental factors (including DIN) covaried more strongly with reach-based spatial structures, and the models suggested that community differences were expressed primarily through diversity-related pathways (α-diversity as a proximal predictor of β-diversity), rather than implying a single DIN-variable causal chain. Considering the riverine lifestyle of *S. punctatus* and its tendency to exploit food resources associated with the river bed (including benthic invertebrates), we suggest a plausible cascade from environment to food to host. Specifically, changes in sediment conditions regulated by dam operations may indirectly reshape gut microbiota by altering benthic food sources and the influx of external microbes, but this hypothesis still requires functional validation.

Overall, our findings suggest that the gut microbiota of native fish such as *S. punctatus* may provide a complementary biological signal of spatiotemporal changes in near-bed habitat conditions in cascade-regulated rivers. These results imply that river management should consider the cumulative effects of dry season impoundment on habitats used by mid-water organisms, including near bed foragers, and the potential role of ecological flow regulation in maintaining the microbial adaptability of such native fish.

## Figures and Tables

**Table 1:** Standardized path coefficients of seasonal PLS-PM models. Path coefficients are standardized. Significance was assessed by bootstrap resampling, and *p* values were adjusted within each seasonal model across structural paths using the Benjamini–Hochberg FDR procedure; * *q* < 0.05, *** *q* < 0.001; ns, *q* > 0.05.

Path	Summer Coef.	Summer *p*	Summer *q*	Summer Sig.	Winter Coef.	Winter *p*	Winter *q*	Winter Sig.
SPA → ENV	−0.571	0.000120177	0.000240353	***	−0.938	4.28074 × 10^−19^	2.56844 × 10^−18^	***
ENV → α diversity	0.786	9.0307 × 10^−6^	2.70921 × 10^−5^	***	0.016	0.970688502	0.998337331	ns
SPA → α diversity	0.439	0.006681991	0.010022986	*	−0.435	0.318921468	0.637842936	ns
ENV → β diversity	−0.052	0.746719726	0.746719726	ns	0.0005	0.998337331	0.998337331	ns
SPA → β diversity	−0.295	0.034386797	0.041264156	*	−0.075	0.750898721	0.998337331	ns
α diversity → β diversity	0.781	7.01164 × 10^−7^	4.20698 × 10^−6^	***	0.842	2.39147 × 10^−11^	7.17441 × 10^−11^	***

## Data Availability

All raw 16S rRNA gene amplicon sequencing data have been deposited in the NCBI Sequence Read Archive (SRA) under BioProject PRJNA1346257. Processed tables (OTU table, taxonomy assignments, and sample metadata) and analysis scripts are available from the authors upon reasonable request.

## Supplementary Materials

The following supporting information can be downloaded at: https://www.mdpi.com/article/10.3390/microorganisms14040745/s1, Figures S1–S3 and Tables S1–S7 are provided in a separate supplementary file. Supplementary Data S1–S4 are provided in a single Excel workbook and include Table S8 (differential abundance summary), the full ANCOM-BC2 differential abundance output tables, per-sample sequencing depth (Effective Tags), and OTU totals after downstream filtering. Supplementary Data S5 provides a summary of sequencing output, sample-level QC statistics, and OTU counts before and after downstream filtering.
