# Supplementary material for: Cascade Dams and Seasonality Jointly Structure Gut Microbiome Biogeography in Saurogobio punctatus
Source: Microorganisms. 2026 Mar 26;14(4):745. doi: 10.3390/microorganisms14040745 (PMC13118653; doi:10.3390/microorganisms14040745)
Supplement: Supplementary file 1 [file microorganisms-14-00745-s001.zip › microorganisms-4162343_supplementary_materials.pdf]

Table S1. Names, geographic coordinates, and longitudinal distances of cascade dams in the Qijiang River. Dam IDs (D1–D9) correspond to the labels in Figure 1. Latitude and longitude are reported in degrees–minutes–seconds (DMS). Δkm (from downstream reference) denotes the along-river (thalweg) distance between adjacent dams, using the downstream adjacent dam as the reference; for the downstream-most dam (D1), Δkm is set to 0. Cumulative distance from D1 (km) is the along-river distance from D1 to each dam, calculated as the cumulative sum of Δkm values. Along-river distances were derived in a GIS environment based on the main-channel polyline; all distances are in kilometers (km).

Dam ID	Dam name	Latitude (°N)	Longitude (°E)	Δkm (from downstream reference) (km)	Cumulative distance from D1 (km)	Notes
D1	Wufu	29°6′29.41″	106°28′54.64″	0	0	Downstream-most dam
D2	Chetan	29°3′42.83″	106°30′26.69″	7.73	7.73	
D3	Qiaoxikou	29°2′33.30″	106°34′20.41″	10.59	18.32	
D4	Dachang	29°1′36.73″	106°38′43.72″	11.28	29.6	
D5	Dahua	28°59′46.39″	106°40′32.99″	6.21	35.81	
D6	Shixikou	28°58′48.86″	106°41′48.01″	9.23	45.04	
D7	Zhutan	28°57′13.74″	106°41′10.65″	6.68	51.72	
D8	Gaishidong	28°55′45.23″	106°42′55.64″	13.26	64.98	
D9	Yangtidong	28°50′8.19″	106°40′31.60″	15.11	80.09	Upstream-most dam

Table S2. Physicochemical properties of the water. Abbreviations: TN, total nitrogen; NH_4^+-N; NO_3^--N; NO_2^--N; TP, total phosphorus; Chl-a, chlorophyll a; DO, dissolved oxygen; T (°C); EC (μS/cm).

Season	Sites	TN (mg/L)	NH_4^+-N (mg/L)	NO_3^--N (mg/L)	NO_2^--N (mg/L)	TP (mg/L)	Chla (μg/L)	DO (mg/L)	pH	Tem (°C)	EC (μS/cm)	COD
Summer	S1	0.4474	0.0826	0.3713	0.0285	0.0350	27.00	7.73	8.17	28.80	436	3.24
	S2	0.3956	0.0110	0.3577	0.0382	0.0267	18.81	8.14	8.12	26.30	433	1.2
	S3	0.4319	0.0846	0.3355	0.0349	0.0288	18.66	8.37	8.18	25.80	422	2.8
	S4	0.4811	0.0479	0.3628	0.0264	0.0229	11.58	8.32	8.19	25.40	434	1.23
	S5	0.5148	0.0882	0.3577	0.0195	0.0312	24.70	8.19	8.23	28.30	417	2.24
	S6	0.367	0.0244	0.3628	0.0143	0.0203	17.26	7.92	8.21	27.20	420	1.45
	S7	0.3878	0.0809	0.3423	0.0130	0.0319	21.93	7.97	8.22	25.40	417	1.19
	S8	0.5045	0.0958	0.3270	0.0150	0.0178	13.04	7.88	8.26	28.30	417	1.54
	S9	0.365	0.0765	0.3611	0.0087	0.0203	12.72	7.67	8.04	29.30	408	0.98
	S10	0.3359	0.0263	0.3577	0.0079	0.0342	11.48	7.73	8.01	28.00	392	1.33
Winter	S1	0.4710	0.1655	0.3491	0.0244	0.0226	7.18	9.18	8.49	16.60	208.5	0.75
	S2	0.5760	0.1514	0.3509	0.0262	0.0211	6.94	8.66	8.53	15.30	206.3	0.78
	S3	0.4555	0.1651	0.3338	0.0296	0.0245	6.40	9.16	8.36	15.20	200.7	0.23
	S4	0.4866	0.1524	0.3406	0.0326	0.0304	7.39	9.49	8.40	14.70	192.9	0.38
	S5	0.3077	0.1561	0.2692	0.0103	0.0214	13.89	10.28	9.01	13.60	190.9	0.84
	S6	0.4127	0.1715	0.2760	0.0181	0.0205	12.12	10.17	8.99	13.10	199.6	1.09
	S7	0.3349	0.1215	0.2437	0.0088	0.0283	21.08	10.30	9.13	13.50	163.9	0.38
	S8	0.3077	0.1201	0.2369	0.0262	0.0201	8.44	10.25	9.07	12.60	156.6	0.14
	S9	0.5527	0.0882	0.1791	0.0224	0.0190	7.19	9.86	9.00	13.50	149.0	0.54
	S10	0.1676	0.0680	0.1689	0.0194	0.0198	24.75	10.19	9.03	12.50	127.5	0.61

Table S3. Two-way ANOVA of season and reach effects on α-diversity of *S. punctatus* gut microbiota.

Diversity index	Factor	F value (effect strength)	*p*-value	Partial η2	Conclusion
**Shannon**	Reach (Site)	3.84	<0.001	36.60%	The spatial effect was stronger
	Season	8.88	< 0.01	12.90%	
**Chao1**	Reach (Site)	6.42	<0.001	49.10%	Both factors were significant (season showed a very large F value)
	Season	52.99	< 0.001	46.90%	
**Faith's PD**	Reach (Site)	6.59	< 0.001	49.70%	The spatial effect was stronger
	Season	42.32	< 0.001	41.40%	

Table S4. Alpha-diversity indices for *S. punctatus* gut microbiota. Indices include Shannon, Chao1, and Faith's PD. Sum = summer; Win = winter.

Sites	Shannon		Chao1		Faith's PD	
	Sum	Win	Sum	Win	Sum	Win
S1	2.82	3.04	734.22	1908.56	93.73	180.97
S2	2.78	2.29	528.95	427.37	70.50	42.01
S3	2.52	8.22	1204.32	3288.69	121.89	286.88
S4	3.01	5.02	1518.79	2814.74	156.20	252.45
S5	6.49	5.26	1654.86	2623.08	170.28	246.70
S6	5.45	8.54	1167.39	2862.74	126.97	267.51
S7	3.34	5.54	814.50	2615.38	88.90	249.99
S8	4.29	5.19	1614.46	1759.04	159.22	177.98
S9	3.25	3.87	1632.94	1909.20	161.96	175.33
S10	3.91	3.98	1319.96	1581.96	143.69	149.81

Table S5. Monte Carlo permutation tests (999 permutations) for environmental fits in seasonal RDA (envfit). Values are r^2, nominal p, and BH-FDR–adjusted q (within each season across fitted vectors). Significance is assessed using *q*: * $q < 0.05$; ** $q < 0.01$; *** $q < 0.001$.

Factors	Sum			Win		
	r^2	*p*	*q*	r^2	*p*	*q*
COD	0.42	<0.01	0.0117*	0.18	<0.05	0.0875^{ns}
Chla	0.4	<0.01	0.0117*	0.37	<0.01	0.035*
DIN	0.4	<0.01	0.0117*	0.54	<0.01	0.035*
DO	0.36	<0.01	0.0117*	0.11	0.117	0.1365^{ns}
pH	0.3	<0.01	0.0117*	0.1	0.166	0.166^{ns}
TP	0.26	<0.01	0.0117*	0.12	0.112	0.1365^{ns}
T	0.08	0.2	0.2	0.21	<0.05	0.0875^{ns}

Table S6. Mantel tests for correlations between physicochemical factors and Bray–Curtis gut microbial community dissimilarity in *Saurogobio punctatus*. Columns for r, nominal p, and BH-FDR–adjusted q (within each season across tested variables) are reported for summer (Sum) and winter (Win). Significance is assessed using *q*: * $q < 0.05$; ** $q < 0.01$; *** $q < 0.001$.

Factors	Sum			Win		
	r	*p*	*q*	r	*p*	*q*
TN	-0.16463	1	1^{ns}	0.254703	0.001	0.0014^{**}
NH_4	0.334914	0.001	0.0012	0.427578	0.001	0.0014^{**}
NO_3	-0.02313	0.623	0.6853^{ns}	0.472463	0.001	0.0014^{**}
NO_2	0.145745	0.001	0.0012^{**}	-0.07419	0.983	0.983^{ns}
TP	0.255405	0.001	0.0012^{**}	-0.0469	0.921	0.983^{ns}
Chla	0.153026	0.001	0.0012^{**}	0.215193	0.001	0.0014^{**}
DO	0.45611	0.001	0.0012^{**}	0.663193	0.001	0.0014^{**}
pH	0.275916	0.001	0.0012^{**}	0.607004	0.001	0.0014^{**}
T	0.692647	0.001	0.0012^{**}	0.709372	0.001	0.0014^{**}
EC	0.65672	0.001	0.0012^{**}	0.759356	0.001	0.0014^{**}
COD	0.441784	0.001	0.0012^{**}	0.164341	0.003	0.0037^{**}

Table S7. MRM results for the effects of spatial and environmental distances on gut microbial β-diversity in summer and winter.

**Season**	**Predictor (term)**	**β (estimate)**	***p***
Summer	Space distance	0.007546	0.014501
	Environment distance	0.010188	0.038704
Winter	Space distance	0.019927	0.020502
	Environment distance	-0.001369	0.907391

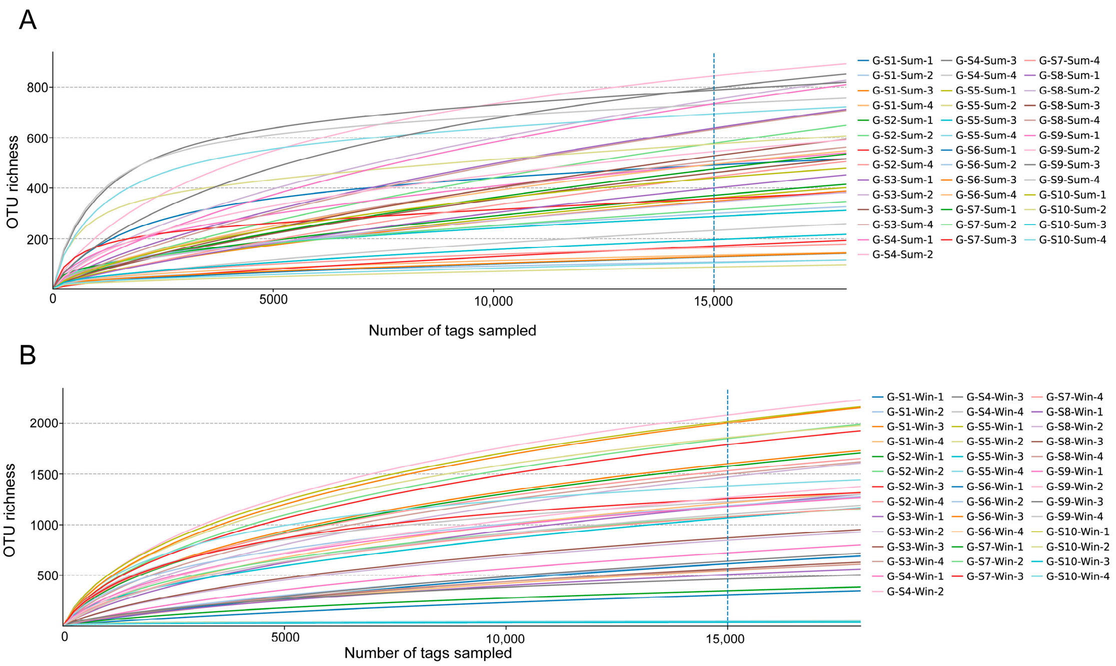

Figure S1. Rarefaction curves of OTU richness in summer (A) and winter (B).

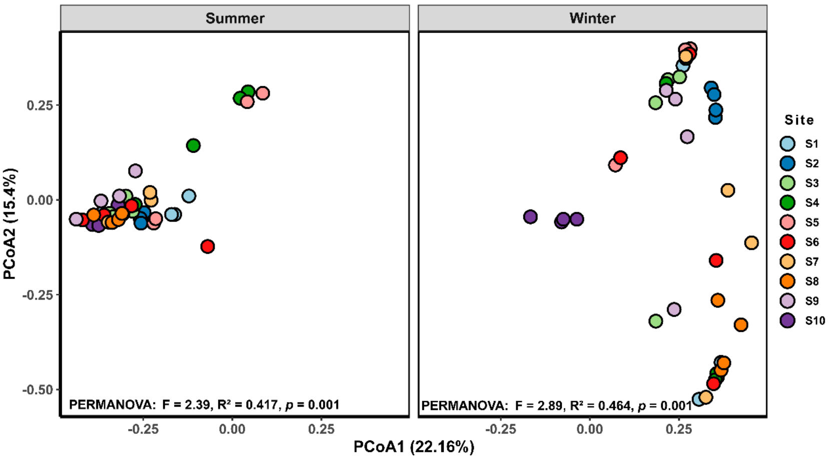

Figure S2. Bray–Curtis PCoA of *S. punctatus* gut microbiota across reaches (S1–S10) in summer and winter. PERMANOVA (999 permutations): Summer F = 2.39, R^2 = 0.417, p = 0.001; Winter F = 2.89, R^2 = 0.464, p = 0.001. Betadisper: Summer p = 0.747; Winter p = 0.631.

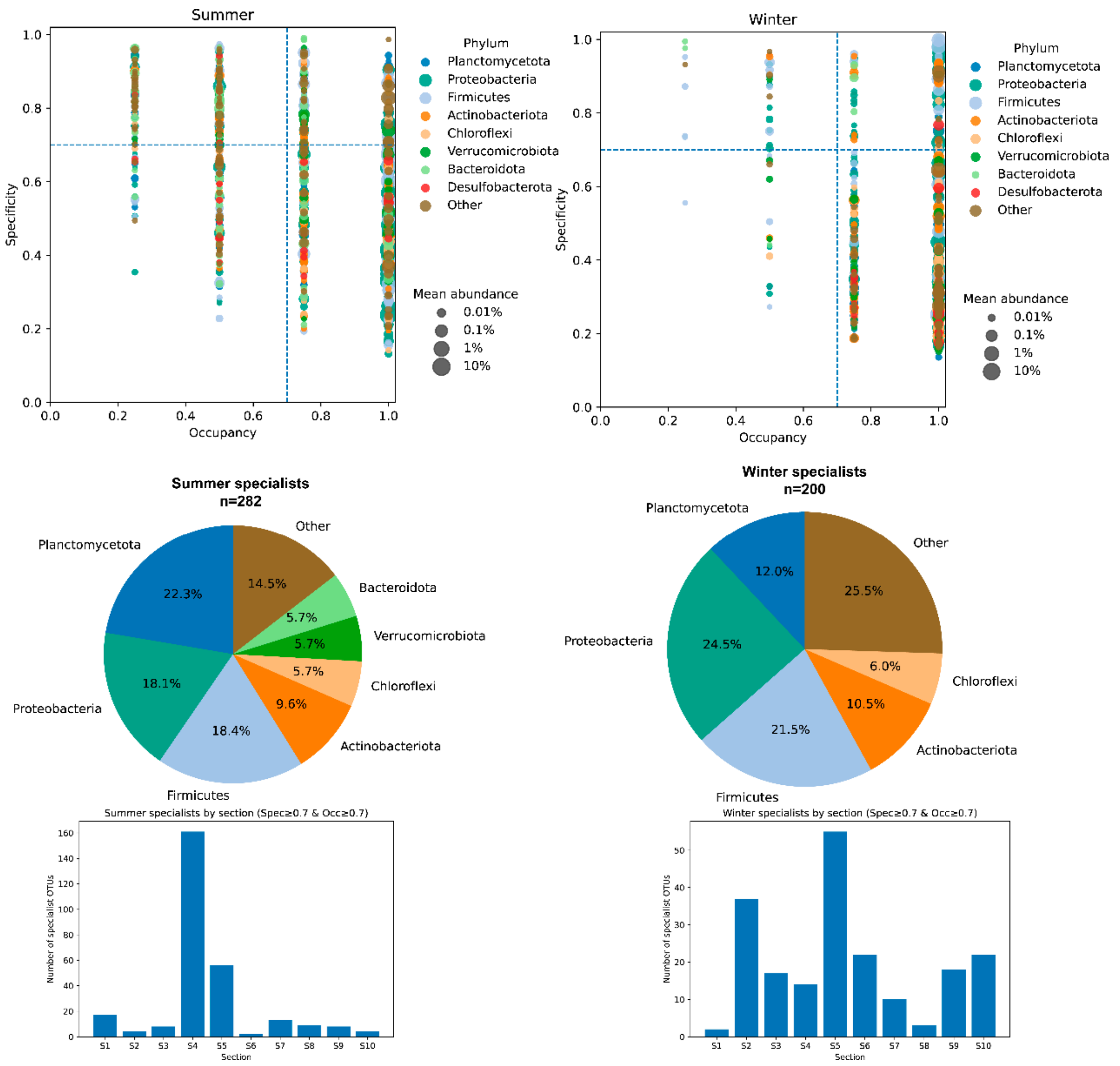

Figure S3. Reach-level SPEC–OCCU patterns of *S. punctatus* gut microbiota in summer and winter. Upper panels show OTU-level specificity (Spec) versus occupancy (Occ) at the reach level, with point color indicating phylum and point size indicating mean relative abundance; dashed lines denote the specialist thresholds (Spec ≥ 0.7 and Occ ≥ 0.7). Middle panels summarize the phylum-level composition of reach-level specialists (Summer: n = 282; Winter: n = 200). Lower panels show the number of reach-level specialists (Spec ≥ 0.7 and Occ ≥ 0.7) in each reach (S1–S10) for summer and winter.
